# Morality and Religion

**Published:** 1865

**Authors:** J. Feeman


### MORALITY AND RELIGION

BY J. FEEMAN, D. D. S.

Morality and religion are two qualifications which are essential to the elevation of the Dental Profession to its proper staudard, and, I very much fear, that if the profession were to-day weighed in a balance, it would be found wanting and suffering more from the neglect of these duties than any other.

A man who is guilty of immorality, to say nothing of his want of religion, is, in my opinion, unqualified to give dignity and character to a profession of such vast importonce; and to my mind there is no doubt but the progress of the Dental Profession has been retarded by unworthy representatives; where it otherwise would have made more rapid strides toward perfection, and might now stand side by side with its sister profession *Materia Medica*, which is only its equal, but yet considered its superior.

The reasons for this sad condition of our profession are plain and numerous. True, a man may give dignity to a profession if he is strictly moral, but without this he will openly disgrace it, and not only disgrace it, but his influence will injuriously affect those who are spending their time and talents for its promotion.

It does appear to me, that no trait in a man's character is more disgusting than profanity, and yet many contract this miserable habit voluntarily, and some really seem to be so ignorant as to believe that it gives them position in society, and consider themselves honored and elevated by its use, and disgraced by its omission.

Was man created with these principles? I answer no. Man was created as pure and perfect as a perfect God could create him, and then left to his own free will to choose either good or evil, and being more prone to choose evil than good, he has, by disobeying and departing from the laws which were given to him to be his future guide and protection, brought upon himself this sad liability to sin. Unfortunately for us, our ancestors departed widely from the path in which it was designed that they should tread; and if we continue in these paths there is danger of our finally being brought to a level with the animal creation, and being left to be guided and controlled by our animal nature.

I therefore believe it to be time for us, as a body of professional men, professing to be men of superior talents and attainments, to set about contriving some method by which such men as these can be detected and prevented entering the Dental Profession.

There has been much said and written upon the necessary qualifications of the dentist. The student must acquire a fine education; he must have at least two years pupilage in some dental office; he must take two courses of lectures in a dental college, and pass a creditable examination in the different branches before he can be an eligible candidate for graduation; but how seldom, how very seldom are any inquiries made as to his moral character, how little is said about this most important feature, and how many times is it passed by entirely unnoticed.

This is one good reason why we have knaves in our profession, and why the profession is ascending so slowly in the scale of moral advancement.

A man who loves and esteems his profession will honor it by yielding implicit obedience to the laws of morality and religion; and the more thorough a man's knowledge is of his profession, of the high and noble sphere in which he is privileged to move, the more sensitive he will be to its prosperity; the more it will affect him to see it desecrated by immorality and drunkenness. We often see men possessing fine natural abilities, in addition to which they have had the advantages of a classical education; have been reared by pious parents, and while surrounded by these heaven-born privileges they were good men, and bid fair to lead lives of dignity and honor; but as soon as they are beyond the boundary of these restraining influences, we find them wandering about and forming their associates among that class of persons whose moral and religious principles have already been shipwrecked; whose life-boat is being tossed to and fro by the angry waves of a troubled sea of vice and wickedness.

It seems that it must be something of a task, for men of fine abilities, who have from infancy been trained and nurtured by pious parents; who have yet echoing in their ears the sayings and warnings of dear departed friends; it seems almost impossible that *such* should turn aside into that door which forms but the first link of an endless chain of vice, all of which will follow in rapid succession and injure its wretched victim both temporarily and spiritually; but still it is so, and, notwithstanding the finer feelings and better judgment of these men, many of them will yield to the tempter's voice; they will launch their mighty intellects upon this foaming, raging sea, and soon they too will be shipwrecked.

The enticements of this class of society are ever ready; their sentinels ever faithful, are constantly walking their beats, ready to welcome an erring brother into their midst; and when the victim once enters their hidden haunt, he finds it much harder to leave than it was to enter, and while there he is so influenced by those around him, that they soon have absolute control and then he is easily led on step by step until he is utterly ruined, and unfit to fill his former position in society; and after this, instead of seeing him walking along your sidewalks, you may find him taking the alleys that lead to this grogshop or that fashionable saloon, in search of the sparkling liquids that will for the time being quiet his guilty conscience.

How deplorable thus to see men of gigantic intellects standing with the intoxicating cup pressed to their lips, and thus, by inhaling their poisonous beverages, destroying all their finer feelings, and under this demoniac influence engaging in all the profanity and vulgarity which man is capable of uttering; nor is this all, fine patrimonies are thus squandered, and men once in easy circumstances become penniless, and finally friendless, walking to and fro in the earth, examples of mighty intellects shattered, and fine judgment dethroned.

It may almost be said of them, they walk to and fro seeking whom they may devour, and while they may not devour us as men, they will, if we have such among *us*, devour our profession, and better devour us than our profession.

Of what value is a fine education, or a towering intellect, if its possessor is not governed by morality, religion, sobriety, honesty and virtue?

Surely without these principles it amounts to nothing; but in connection with them, a finely cultivated mind is one of God's noblest gifts to man; it will ennoble and elevate him; it will give him dignity and character in his profession, and in society, and he will in turn impart the same to the society in which he moves, and to the profession to which he may belong.

Such a man as this will adorn the Dental Profession, and may become a guide to others in the profession who may not have been surrounded with as good privileges as he was. Yes, he may be likened unto the star that stood over the manger in which Christ the Savior of the world was born; like this star he may stand over the Dental Profession, and become its saviour.

Yea, we may all be shining stars, if we will only make the proper effort, and live up to the laws of rectitude and honor; and by this means we will soon elevate our profession much nearer its proper standard.

It becomes us to consider the demands of the age in which we live; the present is a remarkable one in our profession; it is distinguished by remarkable events, which promise either the elevation or depression of our profession for coming ages; and when we consider that we are fast playing our part in the great drama of human life, and will soon be called to pass away, that time is fast bearing us onward; when we find that every day is consigning some one to the narrow home appointed for all the living; and in a short time those around us must take our places; stand where we do; act and move in our stead. When we think of this it behooves us to remember how much depends upon our present movements for the elevation of the Dental Profession throughout the entire world.

The question here arises, What shall we do? The time has come when we want men who can and will govern themselves; men actuated by exalted and ennobling principles—not designing men, whose only attachment to the profession consists in the pecuniary benefits they derive; but we want those who truly love the profession; who value truth above error; whose fidelity has not had its origin and growth in the policy and selfishness of alienated hearts; but has been grown in the soil of honor and virtue, and breathed a different air; thus escaping the miasmatic, the noxious effluvia in which vice and dissipation live.

Thus, with honest hearts; in a pure atmosphere; let us share the rich blessings of general intelligence and heavenly virtues—let these grow and mature around and about us, then let polluted, depraved demogogues thrust at us their ire; and we will meet them with red hot balls of truth, until their rage is quelled, or they are driven from the profession. Let us not stand still to see the salvation of our profession, but put our shoulders to the wheel, and do our duty not only to the profession but to the rising generation.

Already the glimmering star appears; the pendulum vibrates; the hand moves on our professional dial plate; the brighter day dawns; the light comes streaming in; ignorance and superstition are waning; the victory will be ours if we prove faithful; and, above all, let us ever remember, that much of what we do, will live after we have left the stage of action. There is no profession or position in life devoid of influence: this influence, this power possessed by men over the destinies of their fellows is not slight; it imposes so solemn a responsibility, that in view of it man may well say, "Who is sufficient for these things?

From the nature of mind, it is impossible that a single thought or act shall be separated from the great web of thoughts and acts which influence those around us, and it is an indisputable fact, that the incentives and motives of daily life are to rule, in a great measure, *the man;* hence, the vast importance of a strictly moral and religious life.

To be, professionally speaking, a great man, let every man first study the inclinations of his own mind, and choose that profession for which he has the strongest preference, and then, by indefatigable diligence, endeavor, to the best of his ability, to adorn that profession. Let no man trust to chance to make him great, because within these bosoms of ours God has created a bed of shining ore, rich as any the sun has ever seen.

God has created us with mighty minds, and as we lift our eyes to the lofty heavens, and survey their glorious tenantry, that sits as a coronet of diamonds upon the brow of night, we feel irresistably, that we are in sympathy with profound greatness, and that we were made for deep thoughts and solemn musings.

There is grandeur in the generous impulses of our nature, and when they are brought in harmony with the greatness and grandeur of the universe—the soul expands and ascends with a bolder flight to higher regions of thought and knowledge.

But there is no object, the conception of which will so elevate and ennoble the mind as that of a Supreme Being.

The knowledge we have of Deity is composed of the richest moral elements, combining whatever is infinite in wisdom, unbounded in benevolence, sublime in power, inexorable in justice, and correct in the exercise of authority.

There can be no true greatness where God is not acknowledged; for that must be the central truth in every mind, around which all other truths must cluster.

To be professionally great and influential we must be truly good; be the friends of God, and of all good men, commune with Nature and through Nature hold a higher and sweeter communion with Nature's God—be in companionship with all that is great and good.

In our religious character, let us be independent of the sneers and jests of others, and in our professional character, be independent and above all temptations to flinch from known duties, and this independence will be the polar star to guide us along the path of usefulness and honor to true greatness.

But without this stability of action, we may follow only that phosphorescent flame which throws a flickering and delusive lustre over the very grave of virtue and honor; but the practice of morality and religion will lead us into paths, so that our age may be as clear as the noon, and as a morning without clouds, and our departure from the world we have honored be like the setting of an autumnal sun, amid the gold and azure that glows upon a gorgeous firmament.

